# Rational use of eculizumab in secondary atypical hemolytic uremic syndrome

**DOI:** 10.3389/fimmu.2023.1310469

**Published:** 2024-01-11

**Authors:** Lucía Cordero, Teresa Cavero, Eduardo Gutiérrez, Hernando Trujillo, Justo Sandino, Pilar Auñón, Marta Rivero, Enrique Morales

**Affiliations:** ^1^ Nephrology Department, Hospital Universitario 12 de Octubre, Madrid, Spain; ^2^ Instituto de Investigación, Hospital Universitario 12 de Octubre (imas12), Madrid, Spain

**Keywords:** eculizumab, complement, renal, atypical hemolytic uremic syndrome, hemolysis

## Abstract

**Background:**

Secondary atypical hemolytic uremic syndrome (secondary aHUS) is a heterogeneous group of thrombotic microangiopathies (TMA) associated with various underlying conditions. Unlike primary aHUS, there is still no hard evidence on the efficacy of complement blockade in secondary aHUS, since the two main series that investigated this subject showed discrepant results. Our work aims to reassess the efficacy of eculizumab in treating secondary aHUS.

**Methods:**

Observational, retrospective, single-center study, in which we analyzed the hematological and renal evolution of 23 patients diagnosed with secondary aHUS who received treatment with eculizumab and compared them with a control cohort of 14 patients. Complete renal response was defined as the recovery of renal function before the event, partial renal response as a recovery of 50% of lost glomerular filtration rate, and hematological response as normalization of hemoglobin and platelets.

**Results:**

We found no statistically significant differences in baseline characteristics or disease severity between both groups. After a median of 5 doses of eculizumab, the group of patients who received complement blockade presented a significant difference in renal response (complete in 52.3% of patients and partial in 23.8%) compared to the control cohort (complete response 14.3% and partial of 14.3%). Rates of hematological remission were similar in both groups (90.9% in the eculizumab cohort and 85.7% in the control cohort).

**Conclusion:**

Early and short-term use of eculizumab in patients with secondary aHUS could be an effective and safe therapeutic option, assuring better renal recovery compared to patients who do not receive complement blockade.

## Introduction

Secondary atypical hemolytic uremic syndrome (secondary aHUS) is a heterogeneous group of thrombotic microangiopathies (TMA) associated with various underlying conditions: drug treatments, systemic diseases, pregnancy, cancer, hematopoietic stem cell and organ transplantation, systemic infections, primary glomerulopathies (1), and monoclonal gammopathies (2). These represent the main cause of TMA and are associated with a high risk of death and progression to kidney failure (3).

In contrast to primary aHUS, where there is evidence of dysregulated activity of the complement alternative pathway (CAP) at the endothelial cell surface (4), in secondary aHUS, there is still doubt whether the CAP has a role in its pathogenesis. A French group analyzed the frequency of complement gene rare variants in a cohort of 110 patients with secondary aHUS, and it turned out to be like those of healthy individuals, concluding that secondary aHUS is not primarily related to complement dysregulation (5). Nevertheless, the absence of genetic abnormalities does not exclude the implication of complement in secondary aHUS. Cumulative evidence suggests that complement hyperactivation also plays an essential role in TMA other than primary aHUS (6–8). The activated procoagulant and proinflammatory phenotype characteristic of TMA can induce a nonspecific activation of complement, which acts as a ‘second-hit’, amplifying the endothelial damage and perpetuating the TMA, rendering it unresponsive to the treatment of the initial cause (8, 9). This theory is supported by *ex-vivo* experimental studies, which demonstrated massive C5b9 deposits in endothelial cells when confronted with the serum of patients with secondary TMA (10, 11).

Until the second decade of the latest century, treatment of aHUS was based on plasma exchange with fresh frozen plasma (12, 13), aiming to remove mutated factors, cytokines, and triggers of endothelial dysfunction and platelet hyperaggregability (14). This treatment improved mortality rates from 50% to 25%, but the effects on renal function were poor, with 50% of patients developing end-stage kidney disease (4). Therapeutic terminal complement blockade at the level of C5 (15) (eculizumab or ravulizumab (16, 17)) have drastically changed the natural history of primary aHUS, reducing mortality and acquired end-stage renal disease (18, 19).

In contrast, in secondary aHUS, to this date, no hard evidence exists of complement blockade usefulness. Several individual cases published in the literature demonstrate a benefit of eculizumab in secondary forms of aHUS (20–25) regardless of the etiology (26, 27), but there are only two main series that aimed to answer this question, and reached two different conclusions: the first from Spain (28) concluded that short treatment with eculizumab can result in rapid improvement in patients with secondary aHUS, and the second from France (5) did not find any difference in hematological or renal remission between patients who received complement blockade and those who did not. Fakhouri’s group, based on current evidence, proposes the use of complement blockade in secondary aHUS depending on the etiology: they recommend it if the trigger is an autoimmune disease with pathogenic role of complement or monoclonal gammopathy (in these cases from the start), and in TMA secondary to drugs or infections (only if TMA persists despite resolution of the cause). When TMA is secondary to malignancy or an autoimmune disease without pathogenic role of complement, they would avoid its use (29).

Given the lack of consensus in this matter, in the present study, we aim to re-evaluate the efficacy of complement-blockade in patients with secondary forms of aHUS comparing the results with a control cohort that did not receive the drug.

## Materials and methods

### Study population

This is an observational, retrospective, single-center study in which we analyzed the evolution of a group of patients diagnosed with secondary aHUS who received treatment with eculizumab (group A) and compared it with a control cohort of patients from the same center diagnosed with secondary aHUS between 1995 and 2019 who did not receive complement blockade (group B).

Inclusion criteria were evidence of TMA, defined by the presence of low platelet count (<150 x 10^9^/L), microangiopathic hemolytic anemia (MAHA), and a precipitating cause listed among the recognized etiologies of secondary aHUS (drug treatments, systemic diseases, pregnancy, malignancy, hematopoietic stem cell and organ transplantation, systemic infections, and glomerulopathies). Exclusion criteria included any other cause of TMA, such as an impaired activity of ADAMTS-13 (or PLASMIC-score > 4 when the latest was not available), the positivity of Shiga toxin, or suspicion of primary aHUS (pathogenic variants in complement genes, previous episodes of TMA, or eculizumab requirement over six months).

All patients treated with eculizumab received anti-meningococcal vaccination, and antibiotic prophylaxis. The treating physician decided the duration of eculizumab therapy based on the patient’s response and individual characteristics. Follow-up was defined as the interval between the onset of eculizumab and the last visit or death. This study was performed in accordance with the Declaration of Helsinki. Given the retrospective nature of the study, we were granted a waiver of informed consent from individual patients.

### Data collection

Baseline and follow-up data were compiled from medical records, following a uniform protocol that included demographics, clinical presentation, cause of aHUS and laboratory parameters that were deemed of interest. All biochemical parameters were analyzed using routine laboratory methods.

Renal biopsies were performed in 10 patients. Histological signs of TMA, number of sclerosed glomeruli, degree of tubular atrophy and interstitial fibrosis and arteriolar wall thickening resembling “onion skin” lesions were recorded in all cases. Except for 3 cases, all samples were evaluated with direct immunofluorescence staining with IgA, IgM, IgG, C3, C4, C1q, Igk, and Igl. In cases where sufficient material was available, an electron microscopy study was also conducted.

Results of the genetic analysis, performed in 13 patients were collected following a common protocol.

Treatments received for hemolytic uremic syndrome, if any, prior to the initiation of eculizumab, as well as the cumulative duration and doses of the C5 inhibitor were collected. Treatment-related complications, as well as progression to end-stage kidney disease or death were also recorded.

### Definitions and outcomes

Complete renal response is defined as the recovery of renal function before the event, partial renal response as a recovery of 50% of lost glomerular filtration rate, and hematological response as normalization of hemoglobin and platelets.

We define the final date of aHUS as the normalization of hematological parameters and stabilization of renal function.

Malignant hypertension was defined as severe blood pressure elevation (commonly >200/120 mmHg) associated with advanced bilateral retinopathy (hemorrhages, cotton wool spots, papilledema), according to 2020 International Society of Hypertension Global Hypertension Practice Guidelines (30).

### Statistical analysis

Statistical analyses were performed with SPSS (version 22) software with two-sided hypothesis testing and a P < 0.05 as the criteria for statistical significance.

Categorical variables were expressed as frequencies and percentages. Continuous variables were expressed as median (minimum and maximum). Categorical variables were compared with Fisher’s exact probability test, and the medians were compared using the Mann–Whitney U test. Multinomial logistic regression was performed to evaluate the relationship between factors (cause of aHUS, its resolution, serum creatinine at the time of aHUS, and use of eculizumab) with the renal response.

## Results

We collected data from 37 patients, 23 belong to group A and 14 to group B.

### Baseline characteristics

We found no statistically significant differences in baseline characteristics between both groups. The median age was similar, 43 years (16, 76) in group A and 43 years (17, 75) in group B, as well as the distribution between genders, with a percentage of men of 39.1% in group A and 42.9% in group B. Regarding medical history, the percentage of hypertensive and diabetic patients was higher in group B (64.3% vs. 47.8% and 35.7% vs. 13%, respectively). The median of serum creatinine was of 1 (0.4, 6.) mg/dl in group A, and 1.4 (0.6, 5.7) in group B, but the difference was not statistically significant (p = 0.61). A higher percentage of patients with chronic kidney disease was found in group A (52.2%), of which there were six transplant patients and two on peritoneal dialysis, compared to group B, where 35.7% of the patients had chronic kidney disease, one with a kidney transplant, and another one on dialysis. Patients in dialysis were not taken into account in the study of renal evolution.

### Causes of aHUS

As shown in **Table 1**, between the 23 patients who received eculizumab, the causes of aHUS were: autoimmune diseases in 4, glomerular diseases in 2, drugs in 9, infection in 4, malignancy in 1, pregnancy in 2, and pancreatitis in 1. In the cohort group, there were 3 cases induced by autoimmune diseases, three by glomerular disease, four by drugs, one by infection, one after transplantation, and two pregnancy related.

**Table 1:** Clinical and biochemical parameters at the time of Ahus.

	Patients treated with eculizumab (n=23)	Patients not treated with eculizumab (n=14)	P
Cause of aHUS, n (%)			
Autoimmune disease	4 (17.3)	3 (21.4)	
Glomerular disease	2 (8.6)	3 (21.4)	
Drug-induced	9 (39.1)	5 (35.7)	
Tacrolimus	6 (26)	2 (14.3)	
Everolimus	2 (8.6)	1 (7.1)	
Carfilzomib	1 (4.3)	0	
Sunitinib	0	2 (14.2)	
Infection	4 (17.3)	1 (7.1)	
Pregnancy	2 (8.6)	2 (14.3)	
Malignancy	1 (4.3)	0	
Other (pancreatitis)	1 (4.3)	0	
**Time to diagnosis, days**	6 (0, 35)	12.5 (0, 60)	
**Age at diagnosis, years**	43.3 (16, 76)	43.8 (17, 75)	0.65
**Sex, female n, (%)**	14 (60.8)	8 (57.1)	1.00
Hypertension, n(%)			
No	5 (21.7)	3 (21.4)	0,80
Grade I	7 (30.4)	0	**0.025**
Grade II	5 (21.7)	1 (7.1)	0.26
Grade III	6 (26.1)	10 (71.4)	**0.005**
Malignant hypertension	2 (8.7)	8 (57.1)	**0.002**
**HemoglobIin, g/dl**	7.3 (3.5, 11.3)	8.9 (4.4, 11.7)	0.12
**Platelets/μcl**	52000 (9000, 1500000)	64500 (15000, 127000)	0.25
**LDH, IU/L**	578 (242, 3813)	789 (297, 5342)	0.21
**Serum creatinine, mg/dl**	5.6 (0.4, 10)	6.9 (1.3, 19)	0.51
**eGFR, ml/min/1.73m^2^**	7 (4, 134)	8 (1, 49)	0.50
**24-hour proteinuria, g/day**	1.76 (0.25, 11.5)	4.09 (0.4, 6.4)	0.067
**Hematuria, n (%)**	13 (72.2)	5 (35.7)	0.33
**Dialysis, n (%)**	12 (52.2)	8 (57.1)	0.67
**Red blood cell transfusión, n (%)**	10 (43.5)	8 (57.1)	0.50
**Platelets transfusión, n (%)**	3 (13)	1 (7.1)	1.00
**Neurological damage, n (%)**	5 (21.7)	2 (14.3)	0.68
**Kidney biopsy, n (%)**	5 (21.7)	5 (35.7)	0.46

CKD-EPI, Chronic Kidney Disease- Epidemiology Collaboration; LDH, lactate dehydrogenase.

### Clinical and biochemical parameters at the time of aHUS

Clinical and biochemical parameters at the time of diagnosis are shown in **Table 1**. Severity of the disease was similar in both groups. Group A reached hemoglobin median of 7.3 (3.5, 11.3) g/dl, 52,000 (9,000, 150,000) platelets/µL, and lactate dehydrogenase (LDH) of 578 (242, 3813) IU/L, with the need for transfusion of blood products in 43.5% of patients, while in group B hemoglobin median was 8.9 (4.4, 11.7) g/dl, 64,500 (15,000-127,000) platelets/µL, LDH 789 (297-5342) IU/L, and 64.2% of the patients required transfusions. The maximum serum creatinine (sCr) reached in group A was 5.6 (0.5, 10) mg/dl, median proteinuria was 1.7 (0.2, 11.5) g/24h, 13 patients had microhematuria, and 52.2% required renal replacement therapy. In group B, maximum sCr was 6.9 (1.4, 19) mg/dl, median proteinuria was 4.1 (0.4, 6.4) g/24h, 5 patients had microhematuria, and 57.1% of patients required renal replacement therapy.

The presence of malignant arterial hypertension was the only significantly different feature between groups (8.7% in group A vs 57.1% in group B) while 7 patients had neurological symptoms (5 in group A vs 2 in group B).

### Kidney biopsies

Kidney biopsy was performed in 10 patients (5 from group A and 5 from group B), whose results are shown in **Table 2**. In most cases, the indication of kidney biopsy was renal function impairment (in some cases associated to proteinuria or microhematuria). Two of the patients were kidney transplant recipients (patient #5 and #12). Regarding histologic results, exclusive findings of TMA were identified in 3 patients (#3, #5 and #35), while other lesions were found in the rest. In some cases, these findings corresponded to diseases that might have triggered aHUS (#2 and #32 lupus nephritis, #26 and #29 mesangial glomerulonephritis and #27 vasculitis).

**Table 2:** Histological description of renal biopsies.

Biopsy/Group	Indication	Histological findings			
		Light microscopy	Immunofluorescence	Ultrastructural study	Diagnosis
**2/A**	Renal impairment and proteinuria	Intense glomerulosclerosis Mesangial proliferation Onion skin and thrombi within arteries	“Full house” mesangial and parietal deposits	–	Lupus nephropathy and TMA
**3/A**	AKI	Ischemic necrosis Endotheliosis Platelet trombi	C3 +/+++	–	TMA
**5/A**	AKI and MAHA in kidney transplant recipient	Ischemic necrosis Platelet thrombi	Negative	–	TMA
**8/A**	Nephrotic syndrome	Nodular mesangial expansion Thickening of capillary walls Endotheliosis	IgM +/-	Thickening of basement membranes Fibrils of 12nm in the mesangium	Diabetic glomerulosclerosis and chronic TMA
**12/A**	Malignant AHT and AKI in kidney transplant recipient	Glomerulosclerosis Interstitial inflammation Platelet thrombi	–	Reduplication of basement membrane	Nehproangiosclerosis TCMR 1A TMA
**26/B**	AKI	Arteriosclerosis and onion skin appearance Mesangial proliferation	–	Endotheliosis Mesangial non-organized deposits	Mesangial glomerulonephritis and TMA
**27/B**	AKI, hematuria, and proteinuria. ANCA+	Extracapillary proliferation Platelet thrombi	Fibrinogen +		ANCA vasculitis and TMA
**29/B**	AKI, hematuria and malignant AHT	Glomerular sclerosis Mesangial expansion Extracapillary proliferation Onion skin appearance	–	–	Mesangial glomerulonephritis and TMA
**32/B**	AKI	Platelet thrombi Endotheliosis Mesangiolisis Double contours Ischemic glomeruli Hematic cylinders and ATN	Positivity for IgG, C3, C1q in mesangium and capillary walls		TMA Lupus nephritis ATN secondary to hematic cylinders
**35/B**	AKI	Mesangial expansion Double contours TNA and interstitial inflammation	Negative	Reduplication of basement membranes Enddothelisis	Chronic TMA

AHT, arterial hypertension; AKI, acute kidney injury; ANCA, antineutrofil cytoplasmic antibodies; ATN, acute tubular necrosis; MAHA, microangiopathic hemolytic anemia; TCMR, T cell mediated rejection; TMA, thrombotic microangiopathy.

### Complement and genetic studies

Genetic and molecular complement studies were performed in 13 patients (11 from group A and 2 from group B). (**Table 3**). None of the patients carried pathogenic variants, although we found variants of unknown significance in two cases (patient #10 with a variant in CFI c.482 + 8C>T, rs371324158, and patient #11 in VTN c.196G>A). In patient #14 three pathogenic variants in the C5, CR4 and VSIG4 genes, which are not directly related to aHUS, but they may confer a certain predisposition to infections constituting a secondary cause of aHUS. There were five patients (#4, #7, #10, #14 and #29) with CFHR3-CFHR1 gene deletion, four of them in heterozygosis and one in homozygosis, but with no concomitant anti-Factor H autoantibodies were detected.

**Table 3:** Complement genetics in patients with secondary aHUS.

ID	Group	Age	Rare gene variants	Categorization	Risk polymorphisms	CVN	Auto AntiFH
**4**	A	37	None		None	Del *CFHR3-CFHR1* (Het)	Neg
**7**	A	37	None		None	Del *CFHR3-CFHR1* (Het)	Neg
**9**	A	61	None		None	Normal	Neg
**10**	A	37	CFI: c.482 + 8C>T, rs371324158 (Het)	VUS	None	Del *CFHR3-CFHR1* (Hom)	Neg
**11**	A	23	VTN: c.196G>A, rs782257958 (Het)	VUS (1)	None	Normal	Neg
**12**	A	41	None		MCPggaac: (Het)	Normal	Neg
**14**	A	45	C5: c.55C>T, rs121909587 (Het)	Not related to aHUS	None	Del *CFHR3-CFHR1* (Het)	Neg
			ITGAX: c.3358_3363del, p.Ala1121Ilefs*42 (Het)	Not related to aHUS			
			VSIG4: c.1190G>T, rs35553694 (Het)	Not related to aHUS (2)			
**15**	A	30	None		MCPggaac: (Het)	Normal	Neg
**16**	A	47	None		MCPggaac: (Het)	Normal	Neg
**18**	A	39	None		MCPggaac: (Hom)	Normal	Neg
**23**	A	41	None		MCPggaac: (Het)	Normal	Neg
**29**	B	22	Non		None	Del *CFHR3-CFHR1* (Het)	Neg
**33**	B	73	None		None	Normal	Neg

### Outcomes

In group A, the cause of aHUS had been previously treated in 87.5% of them and successfully in 62.5%. The median time from onset of TMA to the start of eculizumab was seven days (1, 35), and the total number of doses was five (1, 12).

Regarding group B, the cause of aHUS was treated in 64.3% of the patients, with success in 57%. Additionally, 57.1% received treatment with plasmapheresis, 35.7% with steroids, 14.3% with rituximab, and 21.4% with fresh frozen plasma.

**Figure 1:**
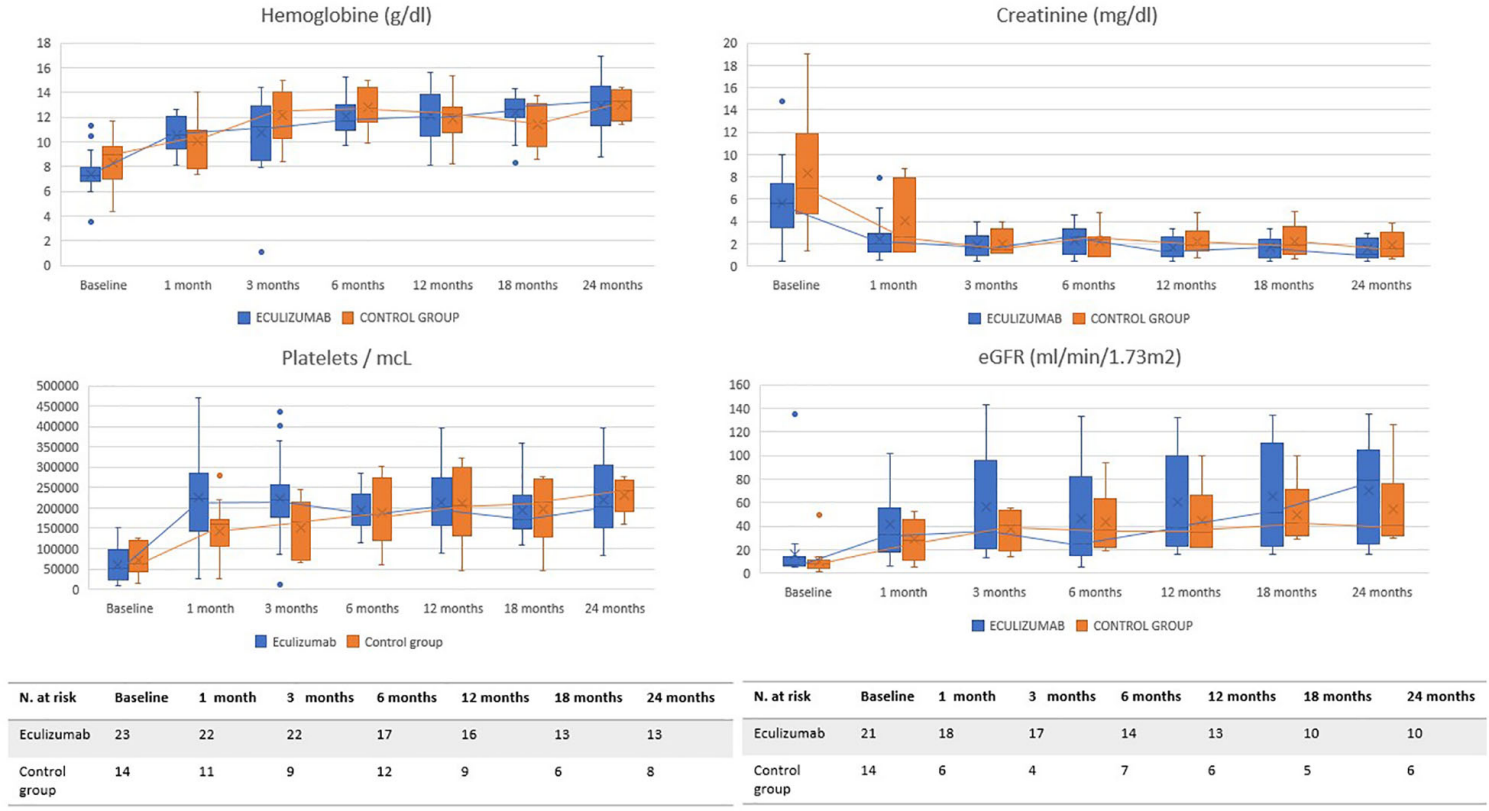
Evolution of main parameters. These graphs show the evolution of the main hematological (hemoglobin, platelets) and renal (serum creatinine and estimated glomerular filtration) parameters during the 24 months after the start of treatment. The blue line represents group A and the orange line group B.

The evolution of the main parameters is exposed in **Figure 1** and **Table 4**. A detailed description is represented in **Supplementary Tables S1** and **S2**. Median follow-up time in group A was 26.9 (0.7, 80.3) months and 35 (0.8, 188) months in group B.

**Table 4:** Main outcomes according to treatment group.

	Patients treated with eculizumab (n=23)		Patients not treated with eculizumab (n=14)	P
**Follow-up, months**	26.2 (0.6, 80.3)		16 (1, 69)	0.40
**Duration of aHUS, days**	33 (5, 73)		51 (6, 368)	**0.032**
**Complete response, n (%)**	9 (39.1)		2 (14.3)	
**Renal response, n (%)** †	No	5 (23.80)	9 (64.2)	**0.014**
	Partial	5 (23.80)	2 (14.3)	
	Complete	11 (52.38)	2 (14.3)	**0.041**
**End-stage kidney disease n (%)** †	6 (27.3)		7 (50)	0.09
**Hematological response, n (%)**	Yes	20 (90.9)	12 (85.7)	0.63
	No	2 (9.1)	2 (14.3)	
**Death, n (%)**	2 (8.7)		0	0.51

aHUS, atypical hemolytic uremic syndrome.

†Patients initially in dialysis were not considered in the analysis of renal evolution.

Rates of hematological remission were similar in both groups (90.9% in group A and 85.7% in group B). In contrast, statistically significant differences were found in terms of renal response with a percentage of complete response of 52.4% and partial response of 23.8% in group A compared to 14.3% complete responses and 14.3% partial responses in group B. End-stage kidney disease was reached in 27.3% of patients from group A vs 57.1% in group B, although this difference was not statistically significant. The duration of aHUS was longer in group B (51 [6, 368] days vs 33 [5, 73] in group A).

After performing multinomial logistic regression, we also found a significant association between the use of eculizumab and complete renal response. This association was not found between the use of eculizumab and partial renal response, and we did not find an association between the renal response and the rest of the studied variables (cause of aHUS, resolution, and maximum serum creatinine reached).

There were 2 patients who died within group A, none of them directly related to secondary aHUS (multiple myeloma, complications of lung transplantation), and no deaths in group B. No adverse events related to eculizumab were found.

## Discussion

In this study, we observed a better renal evolution in patients who received complement blockade.

Before our study, two relatively large series had evaluated the outcomes of eculizumab treatment in this context. The first from Spain (28) included 29 patients with severe secondary aHUS who had received eculizumab, obtaining a rapid TMA resolution in 68% of cases and ≥50% decrease in serum creatinine levels in 51%. Eculizumab was soon discontinued, without aHUS relapses, except in two cases where complement pathogenic variants were identified. Results of the current study are in line with those proposed in the aforementioned series, but in this case comparing the results with a control group without complement inhibition therapy. Of note, some of the cases of our work were already published in the series of Cavero et al (28).

The second series from France (5) included 110 patients with secondary aHUS, of which 35% received eculizumab. The remaining received plasma exchanges, plasma infusions, steroids, or cyclophosphamide (the latter to treat the underlying cause). The two groups had similar rates of hematological remission, chronic kidney disease (stages 3-4), and end-stage renal disease. There are two main reasons which could explain the discrepancy between this study and ours. First, in the French study, patients who received complement blockade presented more severe forms of TMA and were more likely to receive dialysis at the time of diagnosis, so comparison with a control group with different characteristics does not rule out the efficacy of the drug. Second, the time that elapsed from diagnosis to eculizumab initiation was different (mean time from diagnosis to eculizumab onset of 24 days as compared to only seven days in our series). As reported in primary aHUS (18), treatment delay may significantly impact renal outcomes. Thus, in severe cases, complement inhibition since diagnosis rather than waiting until the underlying cause is corrected may be worthwhile.

It should be noted that like the French series (5), hematological response was satisfactory in all patients, regardless of eculizumab treatment status. This might be explained by the fact that hematological evolution is not as catastrophic as renal evolution, even in the pre-complement inhibitors era, as suggested in an Italian series in which hematological remission was achieved with plasma therapy in 58% of patients, while two-thirds progressed to end-stage kidney disease (31).

A key aspect from our study is the limited number of eculizumab doses we have used to reverse TMA expression, confirming previous observations (28) suggesting that only a short course of complement inhibition therapy might be enough. Considering the high cost of C5 inhibition (32, 33), it is essential to rationalize it’s use. Of note, two patients who were already dialysis dependent received eculizumab. One was a kidney transplant recipient due to an aggressive IgA nephropathy who had restarted peritoneal dialysis after graft loss secondary to rejection. He received eculizumab in the context of malignant arterial hypertension and severe neurological symptoms (posterior reversible encephalopathy syndrome). The second patient received eculizumab because of severe anemia and with the aim of preserving residual renal function since the patient had recently started peritoneal dialysis.

Regarding patients’ survival, the 2 deaths observed in the group of patients who received eculizumab were not directly related to the TMA episode but to a severe condition favored by the underlying disease, and there was no case of relapse after discontinuation of eculizumab. Finally, the absence of adverse events related to complement inhibition endorses the previously observed safety of eculizumab.

Our study has several limitations. First, this is an observational and retrospective study, with a small number of patients, especially in the control cohort. Nonetheless, no results have been published to date from any prospective study evaluating the efficacy of complement inhibition in secondary aHUS. Last year, Alexion Pharmaceuticals (Boston, MA, USA) launched a phase 3 randomized, placebo-controlled clinical trial to evaluate the efficacy of ravulizumab in some secondary TMAs (NCT04743804), whose completion is estimated in July 2023.

On the other hand, the study group is very heterogeneous, not only in the underlying aHUS etiology but also in the established treatment, since using eculizumab in secondary aHUS remains empirical and varies between clinical practices.

Finally, the distinction between primary and secondary aHUS is not absolute. TMA is a continuous spectrum of overlapping entities where predisposing genetic factors interact with etiological or triggering factors, resulting in endothelial damage perpetuated by the activation of the complement cascade (26). In our cohort, among the 13 patients who underwent genetic study, two of them carried variants of unknown significance, one carried pathogenic variants that may confer predisposition to infections, and five had CFHR3-CFHR1 gene deletion without anti-Factor H autoantibodies. Since FHR1 helps to combat infection by out competing FH at the bacterial surface (34), it makes some sense to see an apparent increased representation of those individuals in a secondary aHUS cohort, i.e. the CFHR3-CFHR1 deletion is not only associated with increased risk of FH autoantibody. We did not carry out a genetic study in all cases, in some of them because they did not continue follow-up in our department, and in many others (belonging to the group that did not receive eculizumab) because the event occurred in the 1990s or 2000s, when the comprehension of complement role in TMA was limited. Therefore, we may have misclassified a case of primary aHUS as secondary, which could have altered the results of the study. However, precisely this overlap between both entities, and complement activation in secondary forms of aHUS, could explain the efficacy of complement inhibition in these cases (26).

In conclusion, early and short-term use of eculizumab in patients with secondary aHUS could be an effective and safe therapeutic option, ensuring a better renal recovery than treating the underlying cause alone or using other therapies.

## Data availability statement

The original contributions presented in the study are included in the article/**Supplementary Material**. Further inquiries can be directed to the corresponding author.

## Author contributions

LC: Data curation, Formal Analysis, Investigation, Methodology, Project administration, Software, Writing – original draft, Writing – review & editing. TC: Conceptualization, Formal Analysis, Investigation, Methodology, Resources, Supervision, Validation, Visualization, Writing – review & editing. EG: Conceptualization, Resources, Supervision, Validation, Writing – review & editing. HT: Conceptualization, Resources, Supervision, Validation, Writing – review & editing. JS: Methodology, Supervision, Validation, Writing – review & editing. PA: Conceptualization, Methodology, Resources, Supervision, Validation, Writing – review & editing. MR: Conceptualization, Data curation, Formal Analysis, Software, Writing – review & editing. EM: Conceptualization, Data curation, Funding acquisition, Investigation, Methodology, Project administration, Resources, Supervision, Validation, Visualization, Writing – review & editing.
