# Supplementary material for: Rational use of eculizumab in secondary atypical hemolytic uremic syndrome
Source: Front Immunol. 2024 Jan 11;14:1310469. doi: 10.3389/fimmu.2023.1310469 (PMC10808527; doi:10.3389/fimmu.2023.1310469)
Supplement: Supplementary file 1 [file Table_1.docx]

**Supplementary table S1.**  **Evolution according to aHUS etiology in group A.**

aHUS, atypical hemolytic uremic syndrome; APS, antiphospholipid syndrome; AZA, azatioprine; Cs, corticosteroides; Cyc, cyclophosphamide; ID, identification; IgAN, IgA nephropathy; IVIG, intravenous immunoglobulins, MMF, mycophenolate mofetil; MPGN, membranoproliferative glomerulonephritis; RTX, rituximab; SCr, serum creatinine; SLE, systemic lupus erythematosus;

| ID | Age (years), gender | Etiology of aHUS | Treatment of the cause | Eculizumab N. of doses | Time from aHUS to eculizumab (days) | Highest SCr (mg/dl)  Dialysis (yes/no) | Latest SCr (mg/dl) | Follow-up (months) |
| --- | --- | --- | --- | --- | --- | --- | --- | --- |
| 1 | 61, female | Drug (tacrolimus) | Drug reduction | 6 | 10 | 4.4 Yes | 2.61 | 38 |
| 2 | 16, female | Autoimmune disease (SLE) | CS + MMF | 6 | 17 | 7.4 Yes | Dialysis | 0.5 |
| 3 | 62, male | Infection (meningococic shock) | Meropenem | 7 | 4 | 14.7 Yes | 2.34 | 25 |
| 4 | 37, male | Other (pancreatitis) |  | 8 | 3 | 8.5 Yes | 1.05 | 37 |
| 5 | 38, male | Autoimmune disease (SLE + APS) | CS + Cyc + RTX | 7 | 1 | 10 Yes | Dialysis | 72 |
| 6 | 27, female | Autoimmune disease (SLE) | CS + AZA | 5 | 6 | 0.4 No | 0.64 | 35 |
| 7 | 37, female | Pregnancy |  | 15 | 2 | 6.7 No | 0.58 | 5 |
| 8 | 50, female | Infection (viral infection in pulmonar transplant recipient) | Supportive therapy | 9 | 8 | 3.6 No | Dialysis | 26 |
| 9 | 61, male | Malignancy (prostate cancer) | Surgery | 8 | 4 | 5.7 Yes | 1.18 | 80 |
| 10 | 37, female | Glomerular disease (MPGN) in hepatic transplant recipient |  | 5 | 19 | Dialysis | Dialysis | 3 |
| 11 | 23, female | Pregnany |  | 6 | 2 | 8 Yes | 0.73 | 27 |
| 12 | 41, female | Autoimmune disease (SLE) | Cs + MMF | 10 | 14 | 4.5 No | 2.4 | 51 |
| 13 | 48, male | Infection (Parvovirus B19) | IVIG | 2 | 35 | 6 Yes | Dialysis | 18 |
| 14 | 45, male | Drug (tacrolimus) | Drug withdrawn | 3 | 7 | Dialysis | Dialysis | 54 |
| 15 | 29, male | Drug (tacrolimus) | Drug withdrawn | 3 | 1 | 4.3 No | 1.02 | 49 |
| 16 | 46, female | Infection (Parvovirus B19) |  | 4 | 7 | 2.2 No | 0.75 | 63 |
| 17 | 76, female | Drug (everolimus) | Drug withdrawn | 3 | 7 | 5.7 No | 1 | 15 |
| 18 | 39, male | Glomerular disease (IgAN) in kidney trasplant recipient) |  | 1 | 1 | Dialysis | Dialysis | 3 |
| 19 | 55, female | Drugs (tacrolimus) | Drug reduction | 12 | 20 | 7.3 Yes | 2.16 | 36 |
| 20 | 66, female | Drug (everolimus) | Drug withdrawn | 2 | 1 | 3.2 No | 1.3 | 12 |
| 21 | 72, female | Drug (carfilzomib) | Drug withdrawn | 3 | 8 | 2.6 No | 1 | 3 |
| 22 | 64, male | Drug (tacrolimus) | Drug withdrawn | 2 | 12 | 2 Yes | Dialysis | 0.5 |
| 23 | 41, female | Drugs (tacrolimus) | Drug withdrawn | 5 | 12 | 5.6 No | Dialysis | 8 |

**Supplementary table S2. Evolution according to aHUS etiology in group B**

aHUS, atypical hemolytic uremic syndrome; ANCA, antineutrophil cytoplasmic antibodies; Cs, corticosteroides; Cyc, cyclophosphamide; FFP, fresh frozen plasma; ID, identification; IgAN, IgA nephropathy; MMF, mycophenolate mofetil; PE, plasma Exchange; RTX, rituximab; SCr, serum creatinine; SLE, systemic lupus erythematosus;

| ID | Age, gender | Etiology of aHUS | Treatment of the cause | Plasmapheresis | Other treatments | Highest SCr (mg/dl)  Dialysis (yes/no) | Latest SCr (mg/dl) | Follow up (months) |
| --- | --- | --- | --- | --- | --- | --- | --- | --- |
| 24 | 30, female | Pregnancy |  | Yes | CS | 5.1 Yes | Dialysis | 1 |
| 25 | 48, female | Drug (tacrolimus) | Drug withdrawn | Yes | CS + FFP | 5.4 No | 1.5 | 60 |
| 26 | 38, male | Glomerular disease (IgAN) |  | No |  | 7.7 No | Dialysis | 69 |
| 27 | 59, female | Autoimmune disease (ANCA associated vasulitis) | Cyc + CS + PE + RTX | Yes |  | 17.3 Yes | Dialysis | 1 |
| 28 | 34, female | Pregnancy |  | Yes | CS + RTX + FFP | 12.7 Yes | 2.1 | 8 |
| 29 | 22, male | Glomerular disease (IgAN) |  | No | FFP | 19 Yes | Dialysis | 8 |
| 30 | 53, female | Drug (sunitinib) | Drug withdrawn | Yes |  | 4.8 Yes | Dialysis | 12 |
| 31 | 21, male | Glomerular disease (IgAN) |  | No |  | 6.4 No | 3.92 | 2 |
| 32 | 17, female | Autoimmune disease (SLE) | Cyc + Cs + MMF | Yes | CS | 4.2 No | 0.77 | 60 |
| 33 | 73, female | Infection (cholecystitis) | Cholecystectomy and antibiotics | Yes |  | 3.5 No | 1.14 | 60 |
| 34 | 51, female | Autoimmune disease (ANCA associated vasculitis) |  | Yes |  | 7.5 Yes | Dialysis | 1 |
| 35 | 75, male | Drug (sunitinib) | Drug withdrawn | No |  | 1.4 No | 2.04 | 35 |
| 36 | 39, male | Drug (everolimus) | Drug withdrawn | No |  | 11.5 Yes | Dialysis | 38 |
